# Engineering an AB_5_ Protein Carrier

**DOI:** 10.1038/s41598-018-30910-y

**Published:** 2018-08-23

**Authors:** Bruce R. Lichtenstein, Birte Höcker

**Affiliations:** 1 Max Planck Institute for Developmental Biology, 72076 Tübingen, Germany; 2 Department of Biochemistry, University of Bayreuth, 95447 Bayreuth, Germany

## Abstract

The promise of biologic therapeutics is hindered by the challenge to deliver their activity to biochemically relevant sites within diseased cells. The favourable application of the natural protein carriers of the AB_5_ toxin family to this challenge has been restricted owing to still unresolved requirements for assembling non-native cargo into carrier complexes. Here, we clarify the properties of fusion peptides which allow co-assembly of a selected fluorescent protein cargo with the non-toxic B subunit of a heat-labile enterotoxin. We establish the influence of sequence length, sequence identity and secondary structure of these linking domains on the assembly and disassembly of the complexes. Through our engineering framework we identify several non-native, reduced length fusion sequences that robustly assemble with the native carriers, maintain their ability to deliver protein cargo to cells, and demonstrate substantially refined *in vitro* properties. Constructs based upon these sequences should prove directly applicable to a variety of protein delivery challenges, and the described design framework should find immediate application to other members of the AB_5_ protein carrier family.

## Introduction

The greatest promise of biologic drugs is to address cellular dysfunction with the introduction of a protein capable of supplementing deficient and disease-causing biochemical processes. However, despite a rapid increase in their availability current clinical biologics are limited in operation to binding to or blocking the activity of extracellular and cell surface receptors^1^. The large gulf between the activity of accessible biologics and the potential of protein therapeutics is substantially driven by the challenge to deliver their biochemical activity to its relevant intracellular environment in targeted cells. Most proteins will not cross membranes spontaneously and endocytosis of proteins bound to the surface of mammalian cells is often detrimental to protein function, requiring escape mechanisms prior to degradation in lysosomes^2^.

The focus on endosomal escape has naturally led to a variety of approaches that effect release of endosomal contents, including linking cargo to principally cationic cell-penetrating peptides^3,4^ or small molecules^5^, liposome carriers^6^, incorporation into and delivery from virus-like particles^7^, and fusions to endotoxins like anthrax toxin^8^ or to supercharged proteins^9^. Aside from virus-like protein approaches, which can be targeted to specific cell surface receptors, most of these carriers first bind non-specifically to the negatively charged surface of mammalian cells before endocytosis – a feature that greatly limits the application of these approaches in whole organisms^10^. Although the efficiencies of endosomal escape can vary widely, once released, the protein cargoes can localize to the cytoplasmic and nuclear compartments. Yet significantly, they have no immediate access to the Golgi and ER affording little means to address the increasingly recognized diseases associated with deficiencies in these organelles^11^.

The bacterial AB_5_ toxins have solved the endosomal escape challenge by hijacking the membrane recycling pathways of mammalian cells^12^. Although details differ between the various classes of the toxin family, the B subunit carriers retrotransport their cargo from early endosomes to the trans-Golgi apparatus after binding to specifically glycosylated targets associated with lipid microdomains. Further transport of the toxin cargo to the ER can take several pathways around or through the Golgi apparatus^13,14^. Ultimately it is thought that the protein cargoes move to the cytosol by the endoplasmic reticulum associated degradation (ERAD) pathway^15^. Once the B subunits release their cargo they persist in the Golgi^16,17^, and B subunits alone self-direct their retrotransport. This process gives native protein cargo direct access to the Golgi, ER, cytosol and nucleus, and makes these natural carriers, with well-established *in vivo* function, an attractive framework to construct a broad set of targeting, protein delivery tools.

**Figure 1:**
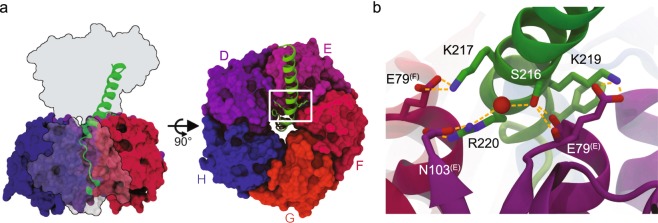
Architectural construction of heat-labile enterotoxin I reveals few, but specific contacts between B subunit carrier units and the A2 subdomain. (**a**) The wildtype LTIB (pdb 1LTI^21^;) is composed of the toxic A1 subdomain (outline), the A2 linking domain (green helix), and the B subunit (colored surfaces, one is made transparent for clarity on left) composed of five identical monomers arranged around a central pore into which the A2 domain penetrates. Specific contacts of interest are highlighted by a box on the right. (**b**) Rotating the view of the white box from (**a**) for clarity, a set of ion-pair interactions and a network of hydrogen bonds (yellow dashes) between the A2 subdomain the B subunit are shown. Interacting residues are labeled including superscript parenthetical chain identifiers for residues originating from the B subunit. Proper structuring of these contacts proves essential for efficient assembly of the cargo-carrier complex, while residues within the pore can greatly enhance the stability of the complex.

The structurally and functionally distinct toxic cargo delivered across the AB_5_ toxin group highlights the advantages of the common non-covalent architecture as an extensible platform for delivery of protein cargo. While direct genetic fusions of protein cargo to the B subunits are possible^18,19^, assembly and glycolipid binding are often inhibited^20^. The relatively simple architecture of the heat labile enterotoxin (LT) family is particularly attractive for further development of modular non-covalent protein carriers. The toxins consist of a homopentameric B subunit that contains a pore into which the C-terminal A2 subdomain of the toxic A subunit cargo is inserted (Fig. 1)^21^. Fusion to the wildtype A2 peptide is sufficient for assembly of the B subunits with non-native cargo during expression^22^. However, once separated cargo and carrier cannot reassemble under native conditions^23^. Thus, the assemblies are kinetically trapped complexes, and the driving forces for assembly and disassembly are likely different.

Application of the toxin family to protein delivery has been mostly limited to vaccine development, e.g.^24^, and, very rarely, non-vaccine protein cargo^25–27^. The limitation is principally due to a lack of detailed understanding of the requirements for co-assembly with generic cargo. Here we report the sequence and structural requirements for efficient and stable co-assembly of protein cargo with the B subunits of heat labile enterotoxin I (LTI), a toxin which is natively expressed in *Escherichia coli* and is closely related to cholera toxin (CT)^28^. We have identified sequence features that control complex stability and show that complexes with varying stability demonstrate markedly different abilities to deliver protein cargo. The methods and insights we describe can be used more broadly with the AB_5_ class of protein carriers, and thus provide a means to improve the *in vitro* properties of complexes with non-native cargo, which is an important milestone towards the creation of a family of robust protein delivery tools.

## Results

### Creation of Expression Cassette

Within the LT family, the A1 toxic subdomain is dispensable for co-assembly of the A2 subdomain with the B subunit^22^, thus making possible the creation of chimeric AB_5_ toxins containing non-native cargoes. Initial attempts to co-express heat labile enterotoxin I B subunits (LTIB) and non-native cargo fused to the native A2 subdomain were complicated by highly inconsistent protein expression levels. This was attributed to expression of the cargo and B subunits in trans from two compatible plasmids. In contrast, the natural gene encoding the holotoxin is polycistronic, with the ribosome binding Shine-Dalgarno sequence (rbs) of the B subunit overlapping the open reading frame (orf) of the A2 subdomain^28^. Inspired by this, we designed a polycistronic gene where the rbs for the B subunits is separated from the orf of the A2 subdomain by a short 8 bp linker. This slight variation on the natural architecture is extensible, granting the use of rbs sites of different strength to control the ratio of the A and B subunits. We selected two established strong rbs sites (AGGAGG/AGGAGA for the cargo/B subunits)^29^, which ensured that like the natural system the cargo is produced in excess over the pentameric B subunits.

We investigated the effect of the periplasmic export sequence on B subunit expression by comparing the signal sequences from DsbA, MalE, OmpA and LTIIb (Supplementary Fig. 1). While we observed no over-expression using the signal sequence from DsbA, MalE proved superior in yielding assembled B subunits over OmpA and the reported high yielding signal sequence from LTIIb^30^. Intriguingly, when expressed alone, the B subunit monomers retained their export sequence (Supplementary Fig. 1). This deficiency in signal sequence removal was not observed when expressing the cargo-B-subunit complexes and possibly reflects the challenge in processing the large quantity of the ‘empty’ B-subunit pentamer produced from these constructs.

To allow independent spectroscopic tracking of the cargo and B subunits, we chose to replace the native, toxic A1 subunit with a fluorescent protein cargo. We selected sfGFP (superfolder GFP) for its stability, brightness, and ability to fold properly in the periplasm when exported using a DsbA signal sequence^31^. The use of sfGFP precludes the need to co-express the TAT translocon required in previous fluorescent protein-B subunit chimeras^25^. Notably, we have subsequently found that B subunits will assemble with cargo in the oxidizing cytoplasms of some *E. coli* strains. A variant of sfGFP(C48A) was prepared to replace a surface exposed cysteine residue to avoid *in vitro* oligomerization. To ease isolation of pure cargo-carrier complex, we introduced an affinity sequence on sfGFP(C48A) and developed a tandem affinity purification strategy described in the Supplementary Information. For the full-length ‘wildtype’ sfGFP(C48A)-LTIA2 chimera, a short glycine rich loop (GSGSG) was installed prior to the last 41 residues of the native LTI A gene product. This slightly truncated A2 sequence was chosen to avert incorporating the biologically necessary C199 that forms a disulfide bond to the native A1 cargo. A gene encoding this construct was cloned between a T7pol promoter region and the rbs site for the MalESS tagged LTIB subunit, thus completing the polycistronic gene assembly (Supplementary Fig. 2). All further variants are based on this construct.

### Design of shortened A2 cargo chimeras

We found that expression of any cargo associated with the full-length A2 subdomain resulted in low yields and evident cell lysis, even when co-expressed with the B subunits. Often the cells would appear red-brown suggesting an adaptive increase in the production of respiratory complexes and hinting that the full length A2 subdomain acts to disrupt the cytoplasmic membrane. When modeled as an idealized alpha-helix, the A2 domain is revealed to have a highly resolved amphiphilic charge distribution across a potentially membrane spanning 35 Å, with a non-polar N-terminus adjacent to a positively charged central region (Supplementary Fig. 3). This arrangement is reminiscent of lytic antimicrobial peptides^32^. In addition, coincident work by Liu *et al*. demonstrating that the A2 domain is capable of penetrating mammalian cells with prolonged 37 °C incubation suggests that biological membrane insertion may be a feature of the wildtype A2 subdomain of LTI^33^. We focused our initial design effort on determining how much of the A2 subdomain is needed for co-assembly with the B subunits to address the poorly behaving ‘wildtype’ (wt) complexes with the idea that eliminating the hydrophobic N-terminal residues might decrease the toxicity evident during expression.

The structures of LTI and the closely related CT (>80% identity) reveal very few specific contacts beyond the interactions between the A2 domain and the pore. Early work by Streatfield *et al*. established that C-terminal truncated forms of the LTI A2 domain lacking almost all of the pore-interacting sequence were capable of accelerating the assembly of the B subunits, resulting in increased yields of the pentamer without forming stable holotoxins^34^. In addition, Tinker *et al*. identified that residue F223 at the opening of the pore in CT, which is preserved in LTI, forms a critical hydrophobic interaction to residues on the B subunit^35^. This phenylalanine residue is within the last turn of the extended A2 helix prior to the loop structure which is embedded within the B subunit pore (Fig. 1). Thus, the orientation ensuring productive contact between the phenylalanine and B subunit residues is established earlier in the helix. This realization led us to re-examine the interface between residues earlier in the A2 sequence and the B subunit. There are several highly specific polar contacts between positively charged residues of the A2 domain and the negatively charged E79 side chains and C-termini of two of the B subunits, in addition to a network of hydrogen bonds centered on a buried water and S216 (Fig. 1b). Such buried polar interactions are known to impart specificity to structural orientation, and we defined the buried serine as the most N-terminal residue of the A2 domain making essential structural contacts. Replacing the serine residue with other amino acids would not preserve the same polar contacts – threonine being an exception. Additionally, due to the compactness of the site very few other amino acids would be tolerated.

We suspected that the helical arrangement of the A2 domain residues interacting directly with the B subunit pentamer would need to be maintained to ensure efficient co-assembly and might be important for complex stability once formed. In the native holotoxin, owing to the large contact area between the A1 and A2 subdomains, the A2 helix is presumably stabilized by interaction with the toxic A1, which we eliminated from our chimeric complex. To increase the helical character of truncated forms of the A2 domain, a helix capping box^36^ was introduced on the N-terminus of the helix for all initial truncations. Since the helix cap on the A2 subdomain would have to be self-contained with no interactions to other protein residues, we sought helical caps that functioned when at least partially solvent exposed, especially the critical hydrophobic staple residues. The sequence was thus selected by manually curating PDB structures with helix caps containing a TPEQ consensus sequence and with solvent exposed hydrophobic staple residues. In generating the shortened versions of the A2 domain, the N-cap sequence LTPEQV was added between the short loop and the helical sequence. N-terminally truncated forms of the A2 domain were designed starting from S216 as the shortest (A2s), and two additional forms, each with an additional turn of the helix (A2m and A2l). Modeling of these variants suggested that the hydrophobic staple residues, leucine and valine, would be presented away from the B subunit, thus preserving native contacts present in the wildtype complex while not introducing additional interactions.

### Variation of Core Residue Identities

In work by Rodighiero *et al*., the higher cellular toxicity of CT over that of LTI could not be explained by enzymatic activity^37^. Rather they found that the toxicity tracked with observed higher detergent resistance of chimeras wherein the CT A2 domain found within the pore of the B subunits was introduced in place of their LTI equivalents. The exact structural basis for this evident difference in detergent resistance was not explored, and it remains unclear if or how the difference in stability manifested as a difference in toxicity. Recent molecular dynamics modeling^38^ of the exchanged sequences within a LTI B subunit pentamer suggested that water accessibility and the energetics of binding are very different between A2 domains of the cholera and LTI sequences. It was proposed that the toxicity difference might be due to structural changes around the conserved ER localization signal found on the membrane associating side of the complex. However, it has been shown that this sequence is not essential for ER localization or toxicity^39^, so this hypothesis cannot be complete. With limited experimental results affirming the basis of the apparent increased stability arising from A2 domain chimeras, we sought to confirm whether the cholera sequence variation (designated with a preceding lowercase c) had an effect on the stability of our non-natural complexes and their delivery. Thus, we generated variants for all of the truncated forms of the A2 domain explored (Table 1).

**Table 1:** Sequences of A2 subdomains in this study.

Full-Length (wt)	NEETQNLSTIYLRKYQSKVKRQIFSDYQSEVDIYNRIRNEL
Short A2 (A2s)	**LTPEQV**SKVKRQIFSDYQSEVDIYNRIRNEL
Medium A2 (A2m)	**LTPEQV**RKYQSKVKRQIFSDYQSEVDIYNRIRNEL
Long A2 (A2l)	**LTPEQV**IYLRKYQSKVKRQIFSDYQSEVDIYNRIRNEL
Short Cholera A2 (cA2s)	**LTPEQV**SKVKRQIFS**G**YQS**DI**D**TH**NRI**KD**EL
Medium Cholera A2 (cA2m)	**LTPEQV**RKYQSKVKRQIFS**G**YQS**DI**D**TH**NRI**KD**EL
Long Cholera A2 (cA2l)	**LTPEQV**IYLRKYQSKVKRQIFSGYQSDIDTHNRI**KD**EL
Medium A2 w/o N-Cap	RKYQSKVKRQIFSDYQSEVDIYNRIRNEL
Short Cholera A2 w/o N-Cap	SKVKRQIFS**G**YQS**DI**D**TH**NRI**KD**EL
Supershort Cholera A2 (cA2ss)	**LTPEKR**QIFS**G**YQS**DI**D**TH**NRI**KD**EL

Residues from the wildtype A2 subdomain sequence observed making contacts to the LTI B subunit are in underline; helix N-capping motifs are bold; core sequence variations associated with CT are emboldened and underlined. The conserved C-terminal ER localization signal sequences RNEL/KDEL are not observed in any reported crystal structure, but they are essential for stable assembly with the B subunits^34^.

### Expression of Fluorescent Cargo-Chimeras

**Figure 2:**
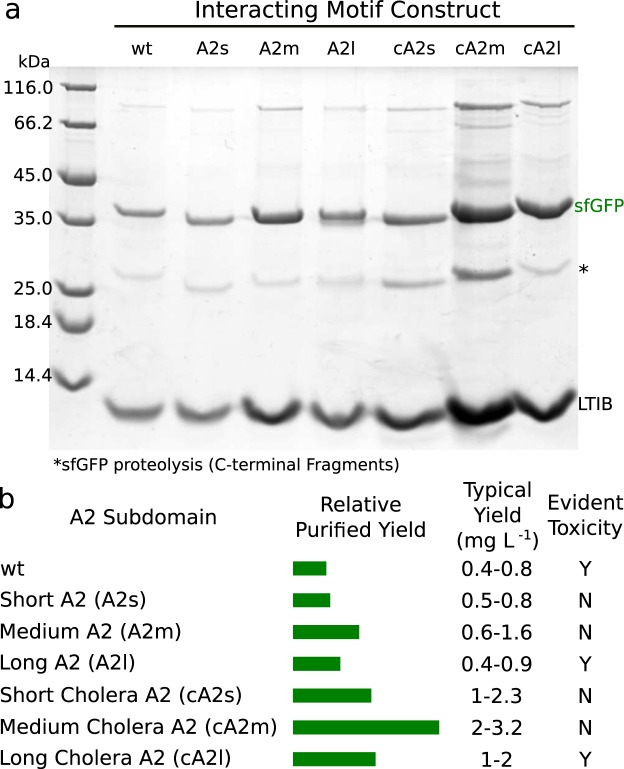
Yields vary markedly between the A2 subdomain variants. (**a**) SDS-PAGE of purified sfGFP-A2:LTIB complexes. The protein bands corresponding to sfGFP and LTIB are labeled. Some sfGFP proteolysis was evident during these purifications (*). Each lane is a sample from identical purifications of four independent expressions for each construct. The purification strategy yields relatively pure and fully assembled complexes. Densitometry of the expressed complexes is compiled qualitatively in (**b**). Typical yields are reported from peak integrals for the purifications of each of the complexes. Toxicity for some complexes was evident through diminishing OD600 after induction, autolysed cell pellets after standard expressions, and substantial quantities of B subunit complexes in the media supernatant of pelleted cells.

Due to the observed toxicity during expression of proteins fused to the wildtype A2, we expressed all variants in the commercial strain NEB T7Express LysY/Iq and used rich defined media (MDAG-11)^40^ for plating and overnight cultures. These precautions made certain that pre-selection against high expressing colonies was minimal and protein expression levels remained consistent. We expressed and purified all of the truncated and cholera sequence variants using our tandem affinity approach. As intended, the fluorescent cargo-A2 fusions expressed in excess over the B subunit pentamers (the ratio of purified B pentamers to sfGFP was 1:4.4 ± 0.3 in a construct with a stop codon introduced between sfGFP and the A2 domain). Under the conditions tested, the longest constructs, wt, A2l and cA2l, showed signs of toxicity, including significantly slower growth post-induction and cell lysis. However, expression of the shorter variants was much better tolerated. To ease quantification of formed complex, we evaluated the yields after identical purifications (Fig. 2). The longer toxic variants expressed poorly compared to the shorter variants (especially variants A2m/cA2m), possibly due to cell lysis prior to exhaustion of the growth media or loss of the expressed protein into the media. In addition, the shortest variants yielded less complex than the middle length variants A2m and cA2m, and all cholera variants expressed better than their native equivalents. This suggests that protein yields are due to a complex mixture of factors: protein toxicity, assembly efficiency, and complex stability. Additional factors such as differential mRNA stability, and differences in mRNA structure near the B subunit rbs are likely present, but given that the observed yield trends repeat for both the native and cholera toxin variants, these are possibly minimized due to the small changes being made between each construct. With the intriguing dependency of the complex yields on A2 sequence, we sought to explore the stability of complexes to elucidate whether these sequence variations had measurable biophysical effects.

### Stability of A2 variant complexes

The fundamental stability of the wildtype complexes has been hinted at being the cause of the different toxicity between CT and LTI. However, this hypothesis remains unexplored and the structural parameters have never been elucidated under native conditions. After purification of the complexes, we noted a significant difference in the complex stabilities upon even overnight incubations at 4 °C, with the shortest complex, A2s, readily decaying into B5 pentamer and free fluorescent-cargo-A2 fusion, which together cannot re-assemble to form the complex. Previous biophysical characterization of this decay process has been challenging owing to the difficulty in separately measuring the B subunit, cargo, and assembled complex. We sought to clarify the structural parameters governing the complex stability by evaluating the kinetics of the decay of the complexes at physiological temperature in the absence of detergent.

**Figure 3:**
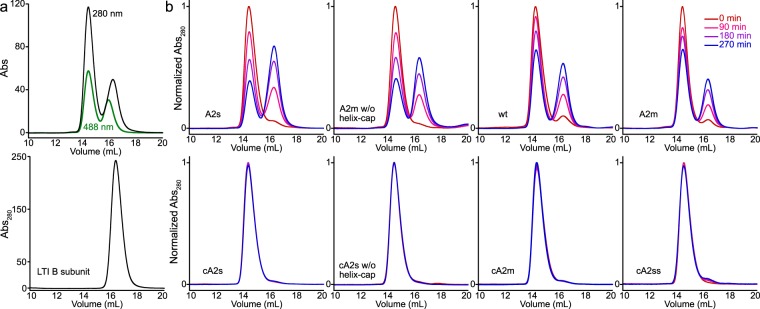
Complex integrity at 37 °C ascertained by gel filtration. (**a**) Unstable complexes decay into two components, sfGFP-A2 was followed at Abs_488_, empty B subunit pentamers at Abs_280_. (**b**) The resolution of complex and B subunit peaks allows for monitoring of complex disassembly. Complex stability tracks with the presence of a helix-capping motif (A2m is more stable than A2m without helix cap and wt) as well as overall length of the sequence (A2s is less stable than wt and A2m). All cholera variants (bottom) decay negligibly over the course of 270 minutes.

We observed large differences in the stability of the complexes when evaluated over several hours at 37 °C (Fig. 3, Supplementary Fig. 4). The cholera sequence variants of all truncations were highly stable within the limits of our detection. In contrast, the non-cholera native variants showed different stabilities depending upon sequence length (A2s was less stable than A2m and wt) and whether the helix was stabilized by a capping motif (a helix cap free variant of A2m and wt were less stable than A2m). Intriguingly, the N-cap free variant of A2m consistently expressed poorly, yielding only enough complex for the decay studies. This suggests that while its stability was between that of the A2s and wt proteins, the lack of a helix cap significantly reduced its ability to assemble with the B-subunits. We took the higher stability of the A2m variant over these other sequences as evidence that the helix cap was working as designed. In addition, it appeared that the stabilizing effect of the cholera sequence variation was dominant over the presence of the N-cap or length of the A2 sequence. To evaluate this, we eliminated the helix capping motif in cA2s and observed that the complex stability did not change. Thus, the helix cap is dispensable for kinetic stability of the complexes. In addition, we prepared an even shorter cholera variant (cA2ss) in which some of the residues involved in polar interactions at the opening of the B subunit pore were replaced with a weaker helix capping motif, and also observed both assembly of the complex and similar high stability. This variant is notable in that residues conserved between CT and LTI A2 domains that make obvious structural contacts to the B subunit can be eliminated with only subtle effects on complex stability. However, these additional variants (cA2s lacking a helix cap and cA2ss) had reduced yields compared to the assemblies with longer and properly helix capped A2 domains, suggesting that the structural features observed at the opening of the pore are more critical for assembly efficiency than complex stability.

### Efficiency of Delivery of the Complexes Tracks with Observed *in vitro* Stability

We sought to understand how changes in the sequence and stability of the sfGFP-A2:LTIB complexes affected cell surface binding and delivery properties of the sfGFP cargo. We chose to use Chinese hamster ovary cell strain K1 (CHO-K1 (ATCC CCL 61)) for the delivery of the sfGFP cargo. Although they do not natively produce the GM1 ligand that LTIB primarily recognizes, the cells are sensitive to LT intoxication owing to the production of simpler glycolipids that LT also binds^41^. While Tinker *et al*. previously described the delivery of fluorescent-protein-wildtype A2 chimera complexes with four members of the LT family, they used eukaryotic cells pre-treated with the receptor ligand to generate an observable signal^25^. We instead sought to reduce the background and clarify the delivery process by using an immunofluorescence (IF) protocol to amplify the signal from the sfGFP cargo. We investigated the binding and delivery capabilities of the wt complex as well as A2s and cA2s, which show the largest difference in stability between complexes of identical A2 subunit length, and thus, served as a means to evaluate *in vitro* stability of the complexes as a factor in their delivery competency.

**Figure 4:**
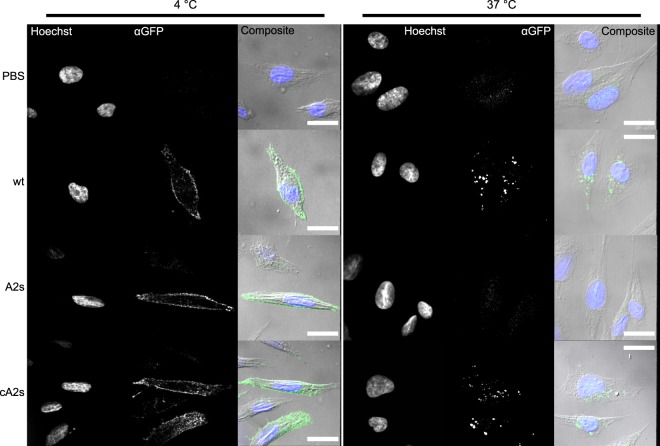
Spinning-disk confocal microscopy of CHO-K1 cells treated with different sfGFP-A2:LTIB complexes reveal variation in delivery competency. Cells were exposed to PBS or ~1 μM of the indicated complexes at 4 °C. After washing they were either immediately fixed or allowed to internalize surface bound complexes at 37 °C. Nuclei were stained with Hoechst 33342 and GFP localization visualized using immunofluorescence with an antibody against GFP. Surface labeling was evident in cells treated with all complexes and fixed at 4 °C (left, single z-slice), while only the more stable complexes wt and cA2s showed consistent juxtanuclear localization of internalized sfGFP cargo after incubation at 37 °C (right, z-stack average). Scale bars in composite frames of the fluorescence channels with DIC images are 20 μm.

At 4 °C, the B subunits are not endocytosed into eukaryotic cells but can bind to their cell surface receptors located in cholesterol-rich lipid microdomains. Application of the complexes at this reduced temperature serves as a means to both confirm surface binding and mediate how much complex is taken into the cells. Once cells treated with the complexes at 4 °C are washed and incubated in media at 37 °C, the surface bound B subunits and any associated cargo are endocytosed and retrotransported to the Golgi. CHO-K1 cells were treated at 4 °C for 30 minutes with approximately 1 μM of freshly purified complexes (Supplementary Fig. 5). After washing, fixation, and IF imaging, the wt, A2s and cA2s complexes all demonstrated the expected surface binding of the CHO-K1 cells in a defined punctate pattern consistent with binding to lipid microdomains (Fig. 4, Supplementary Fig. 5). Differences between the efficacy of the delivery complexes became apparent when cells treated with the complexes at 4 °C were washed and incubated further at 37 °C for 90 minutes, which allows for internalization of surface bound B subunits. Cells treated with both the stable cA2s and moderately stable wt complexes revealed a similar internalized juxtanuclear pattern consistent with Golgi delivery of sfGFP. In contrast, delivery was absent or greatly diminished in CHO-K1 treated with the highly labile A2s complex (Fig. 4, Supplementary Fig. 6). All of the complexes showed similar patterns of cell surface labeling at 4 °C implying that their implicit affinity to the cell surface glycolipids was not disrupted in changing the A2 sequence. Thus, the correlation of delivery efficiency with the stability of the complexes suggests that the A2s complex is dissociating during the retrotransport process. It is unlikely that A2s completely decayed by the same mechanisms in the stability analysis as the kinetics observed for the *in vitro* decay at 37 °C indicate that most of the complex should still be intact after 90 minutes (Fig. 3). Instead, while the complex decay was observed in dilute solutions, the crowded environment of the cell and the active transport process may be sufficient to increase viscous drag forces felt by the complex and thus increase the decay rate.

## Discussion

The AB_5_ toxins show a great potential for application as naturally derived cell targeting and protein delivery tools. The previously used full length wt A2 sequence, which is sufficient for assembly of the LTIB protein carriers and delivery of non-native cargo, comes with toxicity and specificity issues. By refining our understanding of what factors drive assembly between the B subunits of LTI and A2 subunits, we address these issues and present improved AB_5_ protein carriers.

We demonstrate that much of the A2 subunit sequence is disposable, and in removing the uninvolved hydrophobic region that normally binds the A2 subunit to the toxic A1 subunit, expression strain toxicity was eliminated. The preservation of alpha-helical structure of the A2 subunit outside of the pore of the LTIB pentamer is essential for stability of the cargo-carrier complex in the absence of pore-interacting sequence variation. Although other factors are likely involved, assembly efficiency *in vivo* between newly expressed B subunits and sfGFP-A2 fusions appears to be driven by how stable the helix is – sequences that can form at least two turns and are properly helix capped consistently yielded the most complex. This is further supported by the fact that introducing the stabilizing cholera toxin core sequence variation in a construct lacking an N-cap yielded only small quantities of a stable complex. Thus the stability of the helix is especially important during the assembly process, and it is likely that the cluster of polar interactions between the helix of the A2 domain and the opening of the pore of the B subunit must be properly oriented to drive association of the A2 domain and the assembling B subunits. Intriguingly, this extends the interface for targeting small molecules aimed at disrupting the assembly of both cholera toxin and heat-labile enterotoxin in the treatment of diseases associated with these toxins.

Given our observations, we suspect that the A2 domain initially associates with B subunit monomers that have dimerized since the critical polar and non-polar contacts at the opening of the B subunit pore span only two adjacent monomers (Fig. 1). Further oligomerization of the B subunits to the final carrier complex is potentially accelerated^23^ owing to the A2 domain masking the negative charges at the B subunit pore opening and the subsequent increase in buried non-polar surface area as the complex assembles. This process drives protein carrier complex assembly once a binding competent, self-structuring, helical A2 domain is introduced. The nature of the associated cargo possibly only matters insofar as its likelihood to disrupt binding of the A2 domain or the assembly of the B subunits.

By introducing sequence variation derived from CT A2, we observed that the assembly efficiency of the cA2m complex with respect to the B subunits is identical to that of the native holotoxin (~50% of assembled B subunits have cargo bound), while shorter versions show slightly diminished complex formation. We also confirmed that these hybrid sequences increase the stability of the complex. This clarifies that the observed variation in cellular toxicity between CT and LTI can unambiguously be explained by differences in complex stability. Indeed, the reduction in stability observed between the short cA2s and A2s complexes manifested in the decreased ability to deliver protein cargo. Complex stability clearly plays an important role in the utility of the B subunit derived protein carrier complexes. Development of other members of this protein carrier class will benefit from focusing on defining and improving this characteristic.

In our initial evaluation of the structural differences between the CT and LTI holotoxins, we noted that the internal interface of the B subunit pores are surprisingly highly charged. Owing to the homopentameric structure of the B subunits, rings of charged residues line the wall of the complex pores. This creates alternating layers of positively and negatively charged side chains that project into the core of the complex. In the structure of CT,^42^ a histidine residue unique to its A2 domain, which is replaced by a neutral tyrosine in LTI, is associated with a cluster of aspartates and arginines. This set of interactions appears to create a sort of electrostatic staple around the histidine side chain. We suspect that this relatively simple architecture accounts for much of the substantial increase in stability in the cA2 complex variants. In line with this, Craft *et al*.^38^ observed in steered MD studies that the electrostatic environment of the histidine significantly reduces the force needed to drive the cholera A2 domain further into the B subunit pore compared to the LTI A2 domain. In our model, disassociation of the cholera A2 domain from the B subunits requires that the histidine side chain be deprotonated, which is restricted both by electrostatic interactions with nearby aspartates and reduced water/proton accessibility in the core of the complex. In contrast, without the histidine, the LTI A2 domain may unravel out of the pore with a substantially reduced kinetic barrier. Other factors are likely involved, including the observed change in geometry of the LTI and CT A2 domains as they enter the B subunit pore, and we hope that on-going structural determination and biophysical characterization of B subunit complexes with the refined A2 domains will clarify the issue further.

The increased stability of the complexes reported here allows for *in vitro* manipulation without fear of losing activity, building on the modularity of the delivery complexes and opening the door to *in vitro* assembly protocols and delivery of chemically modified or non-biologic cargoes. There does not appear to be a natural sequence or size constraint on the complex assemblies since native B subunits are capable of delivering vesicle-scale loads^43^. Because of their self-structuring, helix cap-driven folding and highly polar sequence, the A2 subunit variants, cA2s and cA2m, can act as fusions to generic cargo to generate delivery complexes with both LTIB and CTB with improved characteristics. The refined properties of these new protein carriers will ease their direct application to the delivery of active cargoes to the Golgi. Future challenges in improving the carriers will have to focus on delivery to different cellular compartments and varying the glycolipid specificity to allow for targeting of select cell-types. The later goal is made more accessible owing to the shared architecture and diverse cell-surface targets of members of the LT family, and the natural engineering of the complexes resolved in this study.

## Methods

### Materials and General Methods

All chemical and materials were used as received from their manufacturer. Routine handling of transformed expression strains were done using MDAG-11 plates and liquid media^40^. D-Galactose agarose was purchased from ThermoFisher or prepared as described elsewhere^44^. Primers were purchased from Sigma-Aldrich or Eurofins, 5′-phosphorylated where needed. DNA sequencing was carried out in-house or by Eurofins. All columns were run on Akta Pure or Purifier FPLCs at reduced temperature. Analytical and small scale preparative gel filtration was performed using GE HealthCare S200 Increase 10/300 GL column on an Akta Pure with a multiple wavelength detector. Protein spectra were recorded on a Varian Bio 50; cell densities were determined at 600 nm on an Eppendorf BioPhotometer. DNA purification kits were purchased from Macherey Nagel. MS/MS analysis from SDS-PAGE gel slices were performed in house using standard protocols. Gel densitometry was performed using Fiji^45^. The genes for heat labile enterotoxin I B subunit and A2 subdomain were optimized for *E. coli* expression and purchased from DNA2.0 (now ATUM) in expression vectors.

### Protein Expression

All expressions were carried out identically. Freshly transformed T7Express LysY/Iq (NEB) were plated on MDAG-11 with appropriate selection antibiotic(s). For each 1 L expression, a single colony was selected and innocluated into 10 mL MDAG-11 media plus selection antibiotic(s), and the culture was allowed to grow overnight at 30 °C. The full volume of the overnight culture was then transferred to 1 L of Terrific Broth (TB) plus kanamycin prepared in a 2.5 L baffled flask and grown with shaking at 30 °C until an OD600 of 0.8–1.2 was obtained. The culture was then induced with 1 mM IPTG at 20 °C overnight (16 hr) with shaking. For large scale preparations, after expression the culture was spun down and processed for protein purification.

### Test Expressions for Signal Sequence Variants

For each signal sequence variant protein expression was carried out as described above. After the overnight induction, the final culture OD600 was noted from a 1/20 dilution of an 2 mL aliquot for each signal sequence variant. Each aliquot was pelleted, washed once with PBS, pelleted again and resuspended in sufficient 2% OTG (w/v, octyl-thioglucoside, Gold Biotechnologies) in PBS to normalize each sample’s cell density based upon the measured OD600. Cells were lysed by gentle rocking in this solution at room temperature (20 °C) for 10 minutes. Clarified lysate was prepared with non-reducing SDS-PAGE sample buffer and split into boiled and un-boiled samples for SDS-PAGE analysis. LTIB is resistant to SDS and only denatures upon heating yielding a simple analysis for determining whether expressed B subunits are assembled into pentamers or are monomeric. Finally, it was noted that OD600 measurements were inversely correlated with apparent over-expression as determined by SDS-PAGE analysis as is anticipated if B subunit expression was resource limiting.

### Protein Purification

#### Large scale tandem affinity preparations

Cell pellets were resuspended (~25 mL/1 L expression) in PBS with 1% OTG (w/v), 1 μg/mL Dnase I, 1 mg/mL lysozyme, 1 mM MgCl_2_, 1x Protease-inhibitor mix HP (1 vial/100 mL, SERVA), and where appropriate BioLock (IBA Lifesciences). After incubation with mixing in this solution at 4 °C for 30 minutes, resuspended cell pellets were mostly lysed, however further sonication (Branson Sonifier 250, medium tip) aided in reducing the viscosity of the supernated and completed the cell lysis. After sonication, cell debris was pelleted, passed through a 0.45 μm syringe driven filter, and loaded onto the first affinity purification column equilibrated with PBS with 0.03% sodium azide on an Akta FPLC system at reduced temperature. After loading and washing the column until the (10 mm) Abs280 dropped below 30 mAU, the bound protein was eluted in a step gradient with reverse flow for 5 column volumes (most protein eluted in the first two column volumes). Subsequently, all fractions containing eluted protein were combined and loaded onto the second affinity purification column pre-equilibrated with PBS-azide. Similarly, after washing the unbound protein off the column (Abs280 less than 30 mAU), bound complex was eluted in a reverse flow step gradient of 5 column volumes. Fractions containing the eluted complex were combined, diluted to 50% glycerol, aliquoted, flash frozen in liquid nitrogen, and stored at −80 °C. Complexes can be stored under these conditions indefinitely. For most purifications, the first column contained 7.5 mL galactose-agarose and was eluted with 500 mM galactose in PBS-azide; the second column was a StrepTrap HP column (GE Healthcare) eluted with 2.5 mM D-desthiobiotin (IBA Lifesciences) in PBS-azide. After protein elution, both columns were regenerated with a reverse flow of 5 CV of 0.5 M NaOH followed by storage under PBS-azide (equilibrated for 5 CV). For careful quantitation of the relative amounts of empty and cargo containing B subunit, the above protocol was adjusted by washing the second column after loading until the Abs280 was stable–this ensured that all of the unbound protein was eluted prior to step gradient.

#### Small scale preparation for cell-culture and time course decay studies

An aliquot of the tandem affinity purified complex was thawed and concentrated to 500 μL using Vivaspin 10 kDa cutoff PES-membrane concentrators (GE Healthcare) to avoid non-specific binding of the complex associated with cellulose membrane concentrators. The sample was loaded onto a S200 Increase 10/300 GL column (GE Healthcare) pre-equlibrated with PBS attached to a Akta Pure FPLC equipped with multiple wavelength monitoring at 280 nm and 488 nm. 0.5 mL fractions were collected, and fractions containing the main peak (generally 2.5 mL), corresponding to fully assembled sfGFP-A2:B subunit complexes as noted by co-incidence of the 280 nm peak and the 488 nm peak, were combined. For cell culture applications, the concentration of the complexes was determined by Abs485 using the reported molar attenuation coefficient of ε_485_ 83300 M^−1^ cm^−1 ^^46^ (Supplementary Fig. 4); if the concentration was too low (<1 μM), the purified complexes were further concentrated.

### Complex Decay Time Course Studies

The combined complex after S200 gel filtration was separated into four 0.6 mL aliquots in Protein LoBind Microcentrifuge Tubes (Eppendorf) previously cooled on ice. Three samples were incubated with gentle rocking at 37 °C. At each time point, 500 μL of each aliquot was applied and resolved with a S200 Increase 10/300 GL column on an Akta Pure monitoring 280 nm and 488 nm. Chromatograms were extracted with PyCORN (https://github.com/pyahmed/PyCORN) and processed with a custom python script which normalized the peak areas for each time point to account for injection variability. Separately, purified LTIB was applied to the S200 Increase column to serve as a control for the elution volume of the B subunit complex (Fig. 3).

### Complex Application and Immunofluorescence

4 × 10^4^ (determined with an Improved Neubauer chamber) CHO-K1 cells grown overnight on 12 mm coverslips in a 24-well plate were washed three times with 4 °C PBS while on ice. 300 μL of PBS solutions of freshly gel-filtrated complexes (approximately 1 μM) were applied to each coverslip and allowed to incubate for 30 minutes at 4 °C (incubations on ice tended to result in a large amount of cell death). Subsequently, the protein solutions were removed and the coverslips washed three times with 4 °C PBS to remove unbound complex. After protein complexes were applied, the coverslips were kept in the dark when not being manipulated.

For surface binding samples, the PBS was removed, and the cells were washed once with 300 μL of ice cold PBS + + (containing 1 mM CaCl_2_ and 0.5 mM MgCl_2_) before being fixed for 10 minutes on ice with 300 μL of freshly prepared ice cold 4% PFA (from 16% PFA, ThermoFisher) in PEM (80 mM PIPES pH 6.8, 5 mM EGTA, 2 mM MgCl_2_). After 10 minutes on ice, fixation was allowed to continue at room temperature for an additional 10 minutes. After fixation, the cells were washed twice for ten minutes with 300 μL PBS plus 50 mM ammonium chloride and stored under 300 μL TBS with 0.03% azide overnight at 4 °C.

For internalization samples, the PBS was removed and pre-warmed DMEM (with HEPES and containing all other supplements as with growth media including 10% FBS) at 37 °C was applied and the coverslips incubated for 90 minutes at 37 °C to ensure complete internalization of surface bound complex. After incubation, cells were fixed at room temperature for 15 minutes as above.

Coverslips under TBS-azide were permeabilized with pre-chilled methanol at −20 °C for 10 minutes in a freezer. The methanol was removed from the coverslips on ice, and the cells were washed three times for five minutes with PBS at room temperature. After aspiration of the PBS, the coverslips were blocked with 5% goat serum (Sigma-Aldrich) in PBS plus 0.1% Tween20 for 30 minutes at room temperature. Coverslips were then treated for 1 hour with the primary antibody in the same solution (Chicken polyclonal IgY αGFP, AbCam ab13970) diluted 500:1. After washing three times five minutes with PBS-tween20, the coverslips were treated for 1 hour with the Alexafluor 488-labeled secondary antibody (Goat Anti-Chicken IgY Alexafluor 488 AbCam, ab150169) diluted 1000:1 in 5% goat serum in PBS-tween20. The coverslips were then washed once for five minutes with PBS-tween20 and incubated for 5 minutes with 1:1000 Hoechst 33342 (ThermoFisher) in PBS. Additional washes 3 × 5 minutes with PBS-tween20 then PBS were followed by mounting on microscope slides with Fluoromount G. The Fluoromount was allowed to cure overnight before sealing the coverslips with clear nail polish and imaging.

### Confocal Fluorescence Microscopy

CHO-K1 cells treated with sfGFP-A2:LTIB complexes and the immunofluorescence protocol were imaged using a 100x/1.4NA oil immersion objective on a custom Leica spinning disk confocal microscope as described elsewhere^47^ equipped with a 405 nm laser for Hoechst 33342 excitation and a 488 nm laser for sfGFP/Alexafluor 488 excitation. Images were processed using Fiji, and image panels were assembled in Affinity Designer (Serif). For images of cells treated and fixed at 4 °C, single z-dimension slices were taken from the middle of the cell most demonstrative of the surface patterning; for those treated at 37 °C, averages of z-stacks were used for the final image.

### Additional Methods

Additional detailed methods are described in the Suppementary Information for cloning and mutagenesis protocols, SDS-PAGE analysis of expressed proteins, cell culture maintenance, and structural modeling of the A2 domain.

## Electronic supplementary material

Supplementary Material

Supplementary Table
