# Supplementary material for: Engineering an AB5 Protein Carrier
Source: Sci Rep. 2018 Aug 23;8:12643. doi: 10.1038/s41598-018-30910-y (PMC6107655; doi:10.1038/s41598-018-30910-y)

Supplementary Information

Engineering an AB_5 Protein Carrier

Bruce R. Lichtenstein and Birte Höcker

Contents **Page**

Supplementary Methods S2

Surface Potential Calculation of Idealized Wildtype A2 Alpha Helix S2
Cloning and Mutagenesis S2
MS/MS Analysis of Incidentally Purified LTIB Subunit without Cargo *S2*
SDS-PAGE Gels S2
Cell Culture S2

Supplementary References S3

Supplementary Figures S4

Supplementary Fig. 1. SDS-PAGE gel analysis of export signal sequence variation on test expressions of heat-labile enterotoxin I B subunits (LTIB). S4
Supplementary Fig. 2. Schematic of expression cassette assembly. S4
Supplementary Fig. 3. Surface potential of an idealized alpha-helical representation of the full-length LTI A2 subdomain calculated with APBS in VMD. S5
Supplementary Fig. 4. Extent of complex assembly as a function of time S5
Supplementary Fig. 5. UV-vis spectra of sfGFP-A2:LTIB complexes in PBS applied directly on CHO-K1 cells. S6
Supplementary Fig. 6. Supplemental microscopy images. S7

Supplementary Methods

Surface Potential Calculation of Idealized Wildtype A2 Alpha Helix

The structure of a straight, idealized alpha-helix with the wildtype A2 sequence was generated with 3.6 residues/turn using backbone dihedral angles of phi -60º and psi -41.12º. The alpha helix was then modeled in VMD[1] (http://www.ks.uiuc.edu/Research/vmd/) using MSMS[2] and the APBS[3–5] plugin with default settings.

Cloning and Mutagenesis

Genes obtained from DNA2.0 were purchased in antibiotic resistance and origin of replication compatible expression vectors (pJexpress414-LTIB (Kan, pUC) and pJexpress434-LTIA2 (Amp, p15A)) behind IPTG inducible T7pol promoter sequences. All cloning was performed using Inverse Fusion PCR Cloning46. Similarly, all mutagenesis followed a protocol similar to the PCR methods described by NEB for Q5 site-directed mutagenesis, with several modifications including eliminating the kinase required for phosphorylating the linear PCR product by using 5' phosphorylated primers. To increase efficiency, only one primer was phosphorylated at each fusion site; when designing the primers the phosphate was selected for 5' A/T nucleotides if present at the fusion site in addition to using 3x dNTPs (NEB) in the PCR reaction. These changes were necessary since Q5 polymerase has a proofreading 3' exonuclease activity that tends to remove T/A nucleotides at the end of PCR products. DpnI (NEB) digestions, where appropriate, were carried out directly in the PCR tube after amplification was complete for 1 hour at 37 ºC. All PCR amplified genes and plasmids were purified by agarose gel extraction. After ligation, chemically competent DH5α were transformed and plated on LB-agar with the appropriate selection antibiotic. Single colonies were selected and evaluated for the presence of the cloned fragment or mutation via colony PCR and DNA sequencing after isolation of pure plasmid via miniprep DNA extraction. Confirmed DNA sequences for all mutants used here are listed in the Supplementary Table.

MS/MS Analysis of Incidentally Purified LTIB Subunit without Cargo

An additional sample of LTIB incidentally purified during cargo-complex purification was treated for SDS-Page analysis similarly to that described in the main text. After SDS-PAGE analysis, Supplementary Fig**.** 1, the protein band associated with the purified and boiled LTIB sample was isolated and extracted with trypsinization. Subsequent sequencing of the obtained fragments by MS/MS yielded 100% coverage of the anticipated full-length B subunit monomer without the export signal sequence.

SDS-PAGE Gels

SDS-PAGE gels were cast using casting blocks for GE Healthcare Might Small II gel chambers using a recipe modified from[7]. Gels were run at constant current (65 mA) or voltage (150 V) using commercially available MES SDS-PAGE (ThermoFisher or VWR) running buffer containing 5 mM sodium bisulfite (Sigma-Aldrich) with stirring of the anode chamber to prevent pH changes at the gel bottom. Gels were stained with blue silver colloidal coomassie [8].

Cell Culture

CHO-K1 (ECACC 85051005, ATCC CCL 61, from Sigma-Aldrich) were grown in Dulbecco's Modified Eagle Medium containing high glucose, phenol red and pyruvate (ThermoFisher) supplemented with 10% FBS (ThermoFisher), 2 mM L-glutamine (ThermoFisher), 100 U/mL penicillin/streptomycin (ThermoFisher), and 1% non-essential amino acids (ThermoFisher) in a 37 ºC incubator with a humidified 5% CO_2 atmosphere in 75 cm^2 culture flasks (Greiner). The culture was split every 2-3 days, maintaining <80% confluency before splitting and less than 20 total passages. After twenty subcultures 'fresh' CHO-K1 were thawed and allowed to recover for at least 3 subculturing cycles before application onto coverslips for microscopy studies. For microscopy, 4 x 10^4 cells (determined

with an Improved Neubauer chamber) were seeded onto pre-washed (3xPBS) 12 mm poly-D-lysine coated Biocoat coverslips (Corning) in the wells of a 24-well plate (Greiner) and grown in the growth media overnight before complexes were applied.

Supplementary References

1. Humphrey, W., Dalke, A. & Schulten, K. VMD: Visual molecular dynamics. *J. Mol. Graph.* **14,** 33–38 (1996).

2. Sanner, M. F., Olson, a J. & Spehner, J. C. Reduced surface: an efficient way to compute molecular surfaces. *Biopolymers* **38,** 305–320 (1996).

3. Baker, N. A., Sept, D., Joseph, S., Holst, M. J. & McCammon, J. A. Electrostatics of nanosystems: Application to microtubules and the ribosome. *Proc. Natl. Acad. Sci.* **98,** 10037–10041 (2001).

4. Dolinsky, T. J., Nielsen, J. E., McCammon, J. A. & Baker, N. A. PDB2PQR: An automated pipeline for the setup of Poisson-Boltzmann electrostatics calculations. *Nucleic Acids Res.* **32,** W665–W667 (2004).

5. Dolinsky, T. J. *et al.* PDB2PQR: Expanding and upgrading automated preparation of biomolecular structures for molecular simulations. *Nucleic Acids Res.* **35,** W522–W525 (2007).

6. Pédelacq, J. D., Cabantous, S., Tran, T., Terwilliger, T. C. & Waldo, G. S. Engineering and characterization of a superfolder green fluorescent protein. *Nat. Biotechnol.* **24,** 79–88 (2006).

7. Sean Moore. Sauer:bis-Tris SDS-PAGE, the very best - OpenWetWare. *Saur Lab Bis-Tris SDS-PAGE Gels* (2013). Available at: https://openwetware.org/wiki/Sauer:bis-Tris_SDS-PAGE,_the_very_best. (Accessed: 6th October 2015)

8. Candiano, G. *et al.* Blue silver: A very sensitive colloidal Coomassie G-250 staining for proteome analysis. *Electrophoresis* **25,** 1327–1333 (2004).

9. Ferencz, C. M. *et al.* Shaping the endoplasmic reticulum in vitro. *Biochim. Biophys. Acta - Biomembr.* **1858,** 2035–2040 (2016).

Supplementary Figures

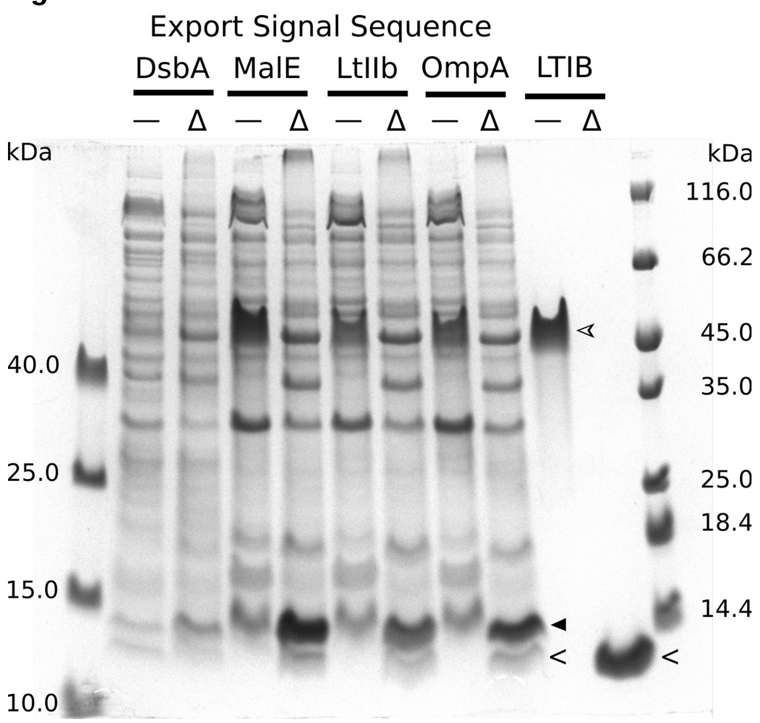

Supplementary Fig. 1. SDS-PAGE gel analysis of export signal sequence variation on test expressions of heat-labile enterotoxin I B subunits (LTIB). Test expressions of LTIB cloned behind various periplasmic export signal sequences (originating from DsbA, MalE, LTIIb, or OmpA) were carried out as described in the supplmentary methods. Samples from test expressions with the indicated signal sequences or from purified LTIB are found below the bold lines; un-boiled samples are indicated with an -- while boiled samples are indicated by Δ. LTIB forms stable, SDS resistant monomers that run with an approximate mass of 45 kDa (indicated by an empty arrow head in the LTIB unboiled lane). Upon heating, B subunits disassociate into their monomers and run at their anticipated size of ~11.7 kD (indicated by the chevrons in both the OmpA and LTIB boiled lanes) or the size of monomers with uncleaved export signal sequences ~14.3 kDa (filled arrow in OmpA boiled lane). The protein band in the LTIB lane was confirmed to be the full-length, signal sequence free B subunits by MS/MS sequencing implying that the evident SDS-PAGE sizes of the protein bands accurately reflect their molecular identities. Over-expression of B subunits alone mostly yields complexes with maintained signal sequences, while co-expression with cargo (the source of the LTIB) gives complexes with the anticipated post-translational proteolysis.

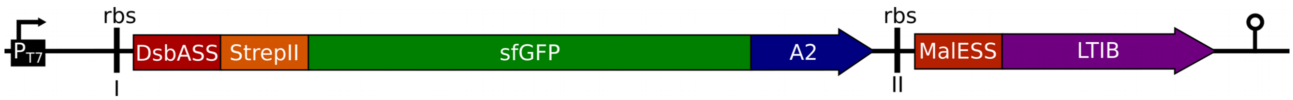

Supplementary Fig. 2. Schematic of expression cassette assembly. The expression cassette for complex formation is a polycistronic gene assembly driven by a T7pol promoter (P_{T7}). Ribosomal binding sites (rbs) I and II are both strong but have distinct sequences, AGGAGG and AGGAGA, respectively. rbs I drives translation of the cargo-A2 fusion described in the text, while rbs II is responsible for translation of the LTI B subunit monomers. In contrast to the native assembly where the second rbs site is within the open reading frame of the A2 subunit, a short 8 bp sequence separates the stop codon for the sfGFP-A2 fusion and rbs II.

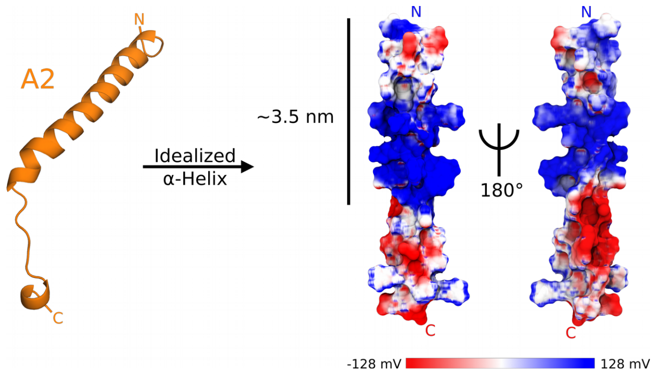

Supplementary Fig. 3. Surface potential of an idealized alpha-helical representation of the full-length LTI A2 subdomain calculated with APBS in VMD. On the left is the structure of the LTI A2 domain as found in the pdb structure 1LTI; the structures on the right are versions of the A2 subdomain as an idealized alpha-helix showing a distinct amphipathic pattern reminiscent of alpha-helical antimicrobial peptides. Surface charges are represented by color gradient scale. The N-terminal, amphipathic and positively charged region is approximately 3.5 nm, or sufficiently long to transverse a bacterial membrane and expressions of proteins fused to shortened A2 subdomains show eliminated expression strain toxicity consistent with removal of the hydrophobic region required for bactericidal effect.

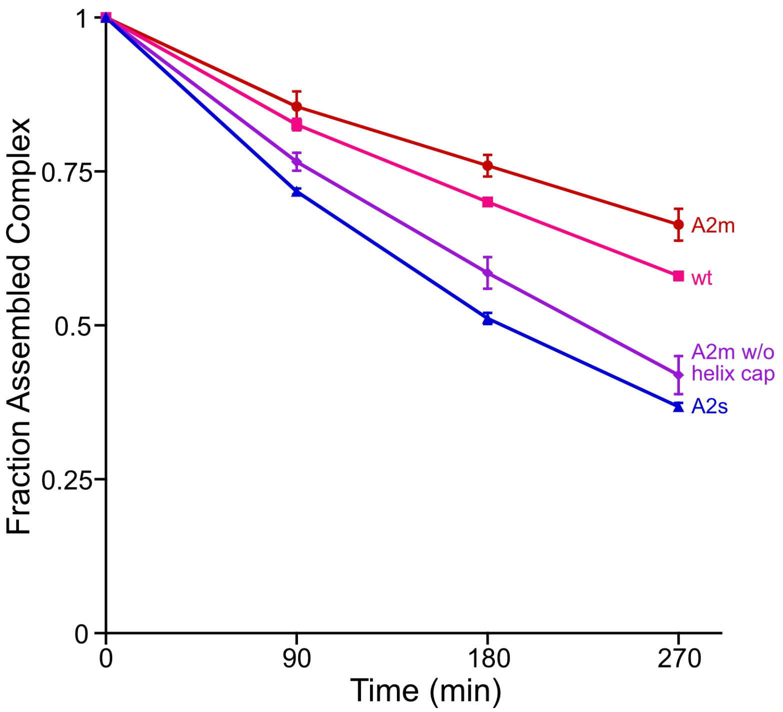

Supplementary Fig. 4. Extent of complex assembly as a function of time (Fig. 3). Assembled complex fraction were determined by integration of peaks associated with the cargo-carrier complex, sfGFP-A2 cargo, and the B subunits using both 488 nm and 280 nm channels. These values were used to derive an average proportion of assembled complex at each time point for the decaying complexes. Displayed errors arise from the standard deviation of these measures. As in Fig. 3, the relative stability of each of the complex is clear, with A2s decaying the fastest and A2m being the most stable of the analyzed non-cholera A2 variants.

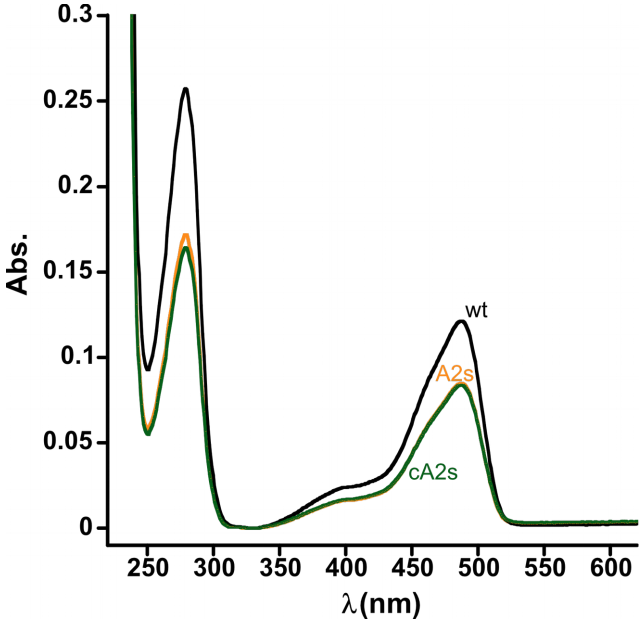

Supplementary Fig. 5. UV-vis spectra of sfGFP-A2:LTIB complexes in PBS applied directly on CHO-K1 cells. Using the reported molar attenuation constant for sfGFP (ε_{485} = 83300 M^{-1} cm^{-1}), the concentrations for wt, A2s, and cA2s were 1.4 μM, 1.0 μM and 1.0 μM, respectively.

Supplementary Fig. 6. Spinning-disk confocal microscopy of CHO-K1 cells treated with different sfGFP-A2:LTIB complexes reveal variation in delivery competency. Cells were exposed to PBS or ~1 µM of the indicated complexes at 4 ºC. After washing they were either immediately fixed or allowed to internalize surface bound complexes at 37 ºC. Nuclei were stained with Hoechst 33342 and GFP localization visualized using immunofluorescence with an antibody against GFP. Surface labeling was evident in cells treated with all complexes and fixed at 4 ºC (left, single z-slice), while only the more stable complexes wt and cA2s showed consistent juxtanuclear localization of internalized sfGFP cargo after incubation at 37 ºC (right, z-stack average). Scale bars in composite frames of the fluorescence channels with DIC images are 20 µm. Figure continues over several pages.

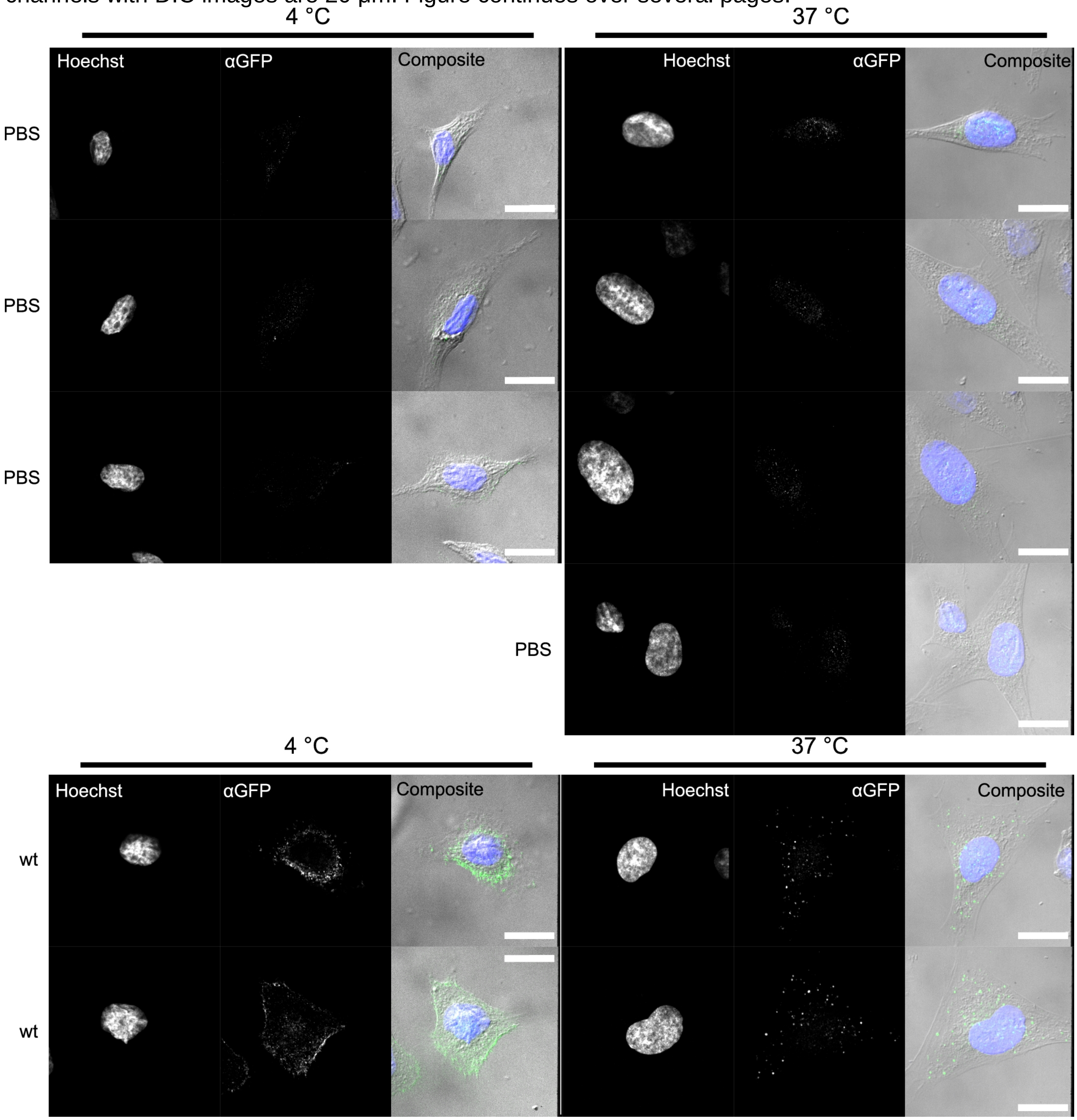

Supplementary Fig. 6 continued.

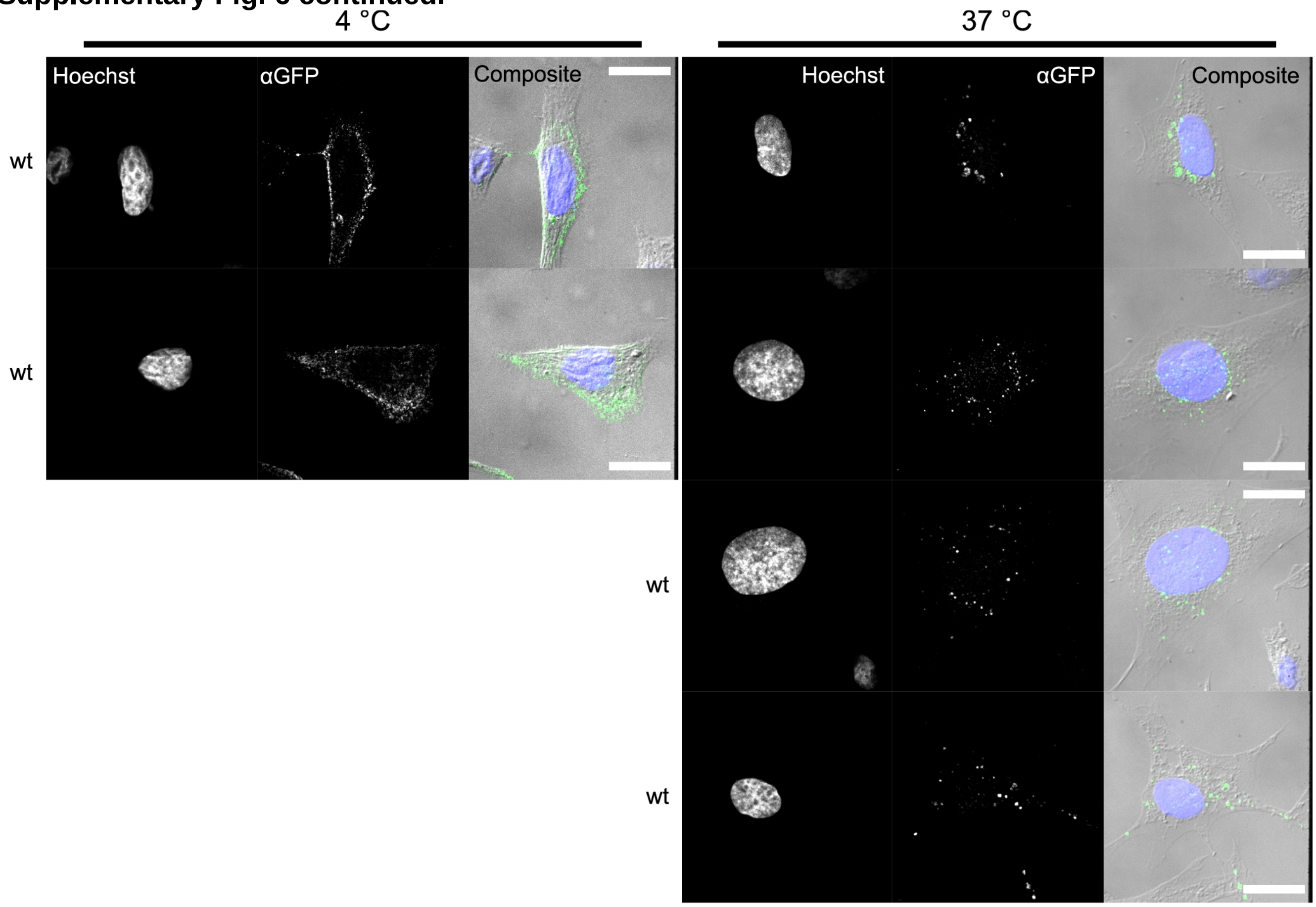

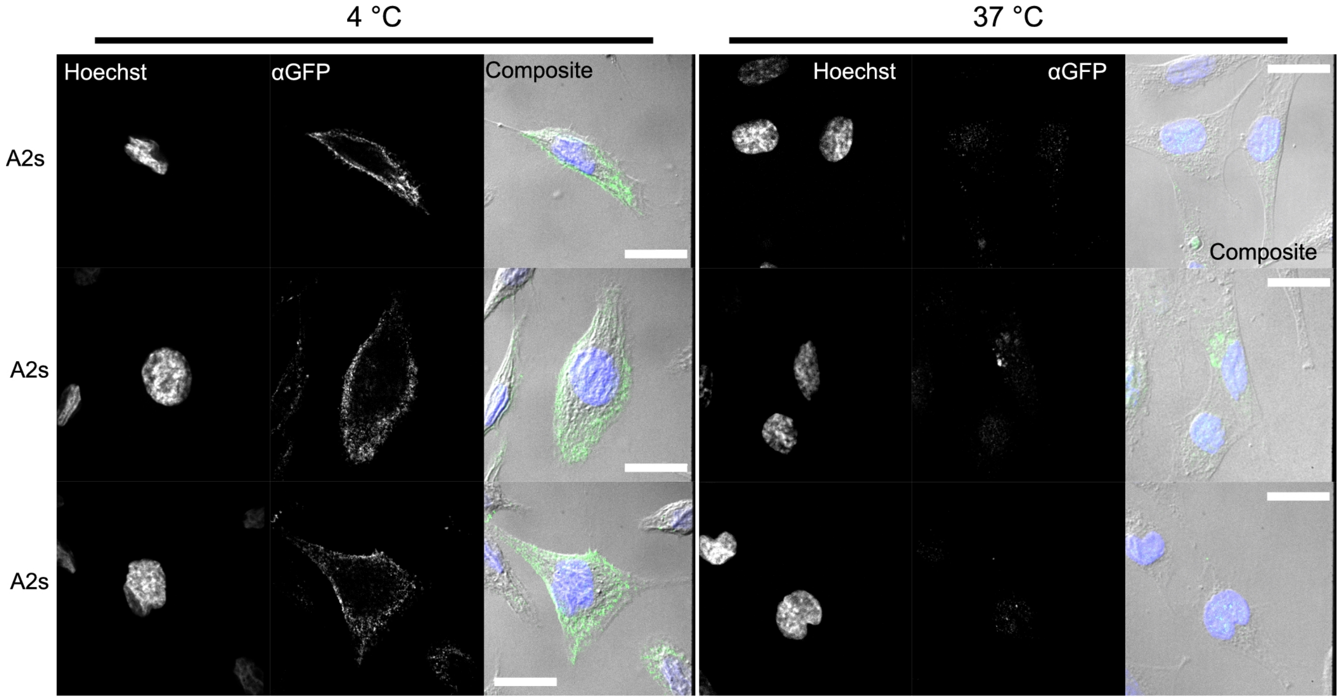

Supplementary Fig. 6 continued.

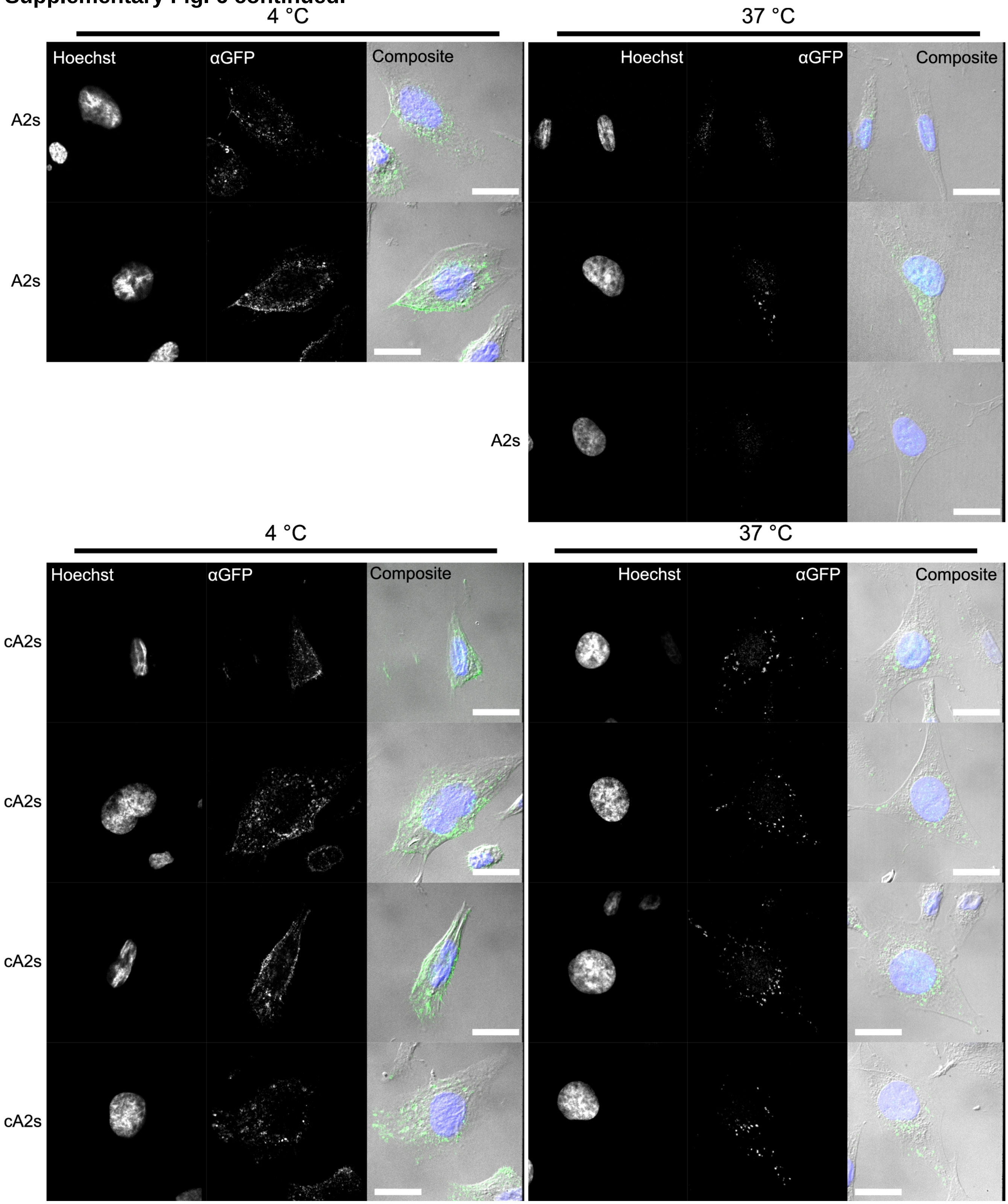

Supplementary Fig. 6 continued.

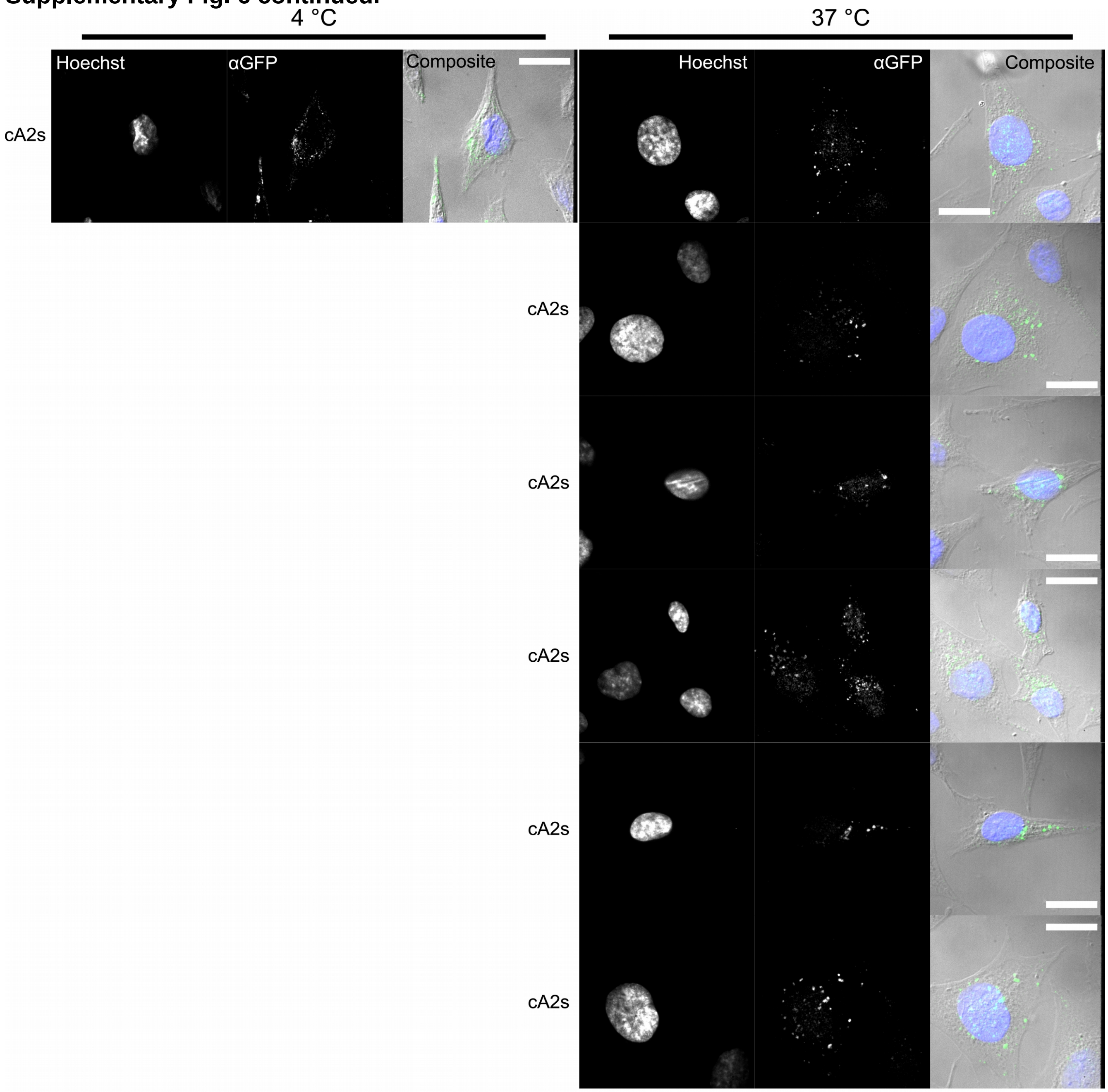

Supplement: Supplementary file 1 — Supplementary Material [file 41598_2018_30910_MOESM1_ESM.pdf]
